# Towards Healthy and Sustainable Diets: Environmental and Nutritional Perspectives

**DOI:** 10.3390/nu18050711

**Published:** 2026-02-24

**Authors:** Georgios K. Vasios, Constantinos Giaginis

**Affiliations:** Department of Food Science and Nutrition, School of Environment, University of Aegean, 81400 Lemnos, Greece

Sustainable diets have been defined as those that are healthful, have low environmental impacts, are affordable, and are culturally acceptable [1,2]. It has recently been clear that the environmental impact of food systems must be reduced to mitigate the effects of climate change and adhere to global climate targets [3,4]. In this respect, sustainable diets are considered a promising pathway towards transforming food systems to support both planetary and human health [5,6]. They promote dietary patterns rich in fruits, vegetables, whole grains, and healthy fats, with moderate or minimal amounts of meat, saturated oils, added sugars, and (ultra) processed foods [7,8]. Several well-known diets, such as the Mediterranean diet, the Nordic diet, Asian diets, vegetarian diets, vegan diets, and others, follow sustainable and healthy dietary patterns [7,8]. Contemporary healthy plant-based food consumption combined with zero-waste and zero-plastic initiatives can reduce the environmental impact of food production systems [9,10]. However, it is highly recommended to investigate whether these different aspects of sustainable diets could manage to exert a positive impact on complex health issues [11,12]. It should also be explored whether the global guidelines on sustainable diets and/or the Sustainable Development Goals (SDGs) may be sufficient to transform regional and local behaviors around the world [13,14]. Hence, future research should be focused on the potential sustainable dietary habits that may change food production, affecting our adaptation to environmental challenges ahead [15,16]. In this respect, the present Special Issue aims to enhance understanding of the interconnection between human nutrition and environmental sustainability following the guidelines of sustainable diets as they should or could be implemented across global, regional, and local scales.

In view of the above considerations, a study by Civero et al. investigated whether consumers may decide to adopt alternative proteins—specifically insect-based, cultured meat, and plant-based options—as part of a transition towards environmentally sustainable diets [17]. This study provided substantial evidence that consumers’ sustainable food intentions may more strongly be shaped by moral identity and the surrounding social context compared to attitudes alone. These findings supported the development of culturally sensitive strategies designed to strengthen moral and normative motivations and foster the adoption of alternative proteins [17]. Another study evaluated the effectiveness of the Memorandum of Understanding (MoU) and assessed the potential impact of decreasing salt in bread on overall salt intake, using the MoU target and the relevant WHO global sodium benchmark [18]. It should be noted that standardizing salt levels can be effectively achieved through legislation, resulting in sustainable long-term benefits. However, this study highlighted the failure of Greece’s voluntary national initiative to reduce salt in non-prepackaged artisanal bread, underscoring the limited impact of non-binding public health interventions. In this respect, this study proposed that a mandatory upper salt limit in non-prepackaged artisanal bread with robust monitoring would represent a more reliable and sustainable strategy for reducing salt content [18].

Furthermore, a prospective, population-based cohort study investigated the associations between educational attainment and 20-year cardiovascular disease (CVD) incidence, mortality, lifetime risk, and burden, and explored the mediating role of healthy and sustainable dietary habits through a sex-specific lens [19]. This study supported evidence that the educational disparities in long-term CVD outcomes may partly be mediated by sustainable dietary habits, highlighting also the need for gender-responsive and equity-focused strategies in cardiovascular prevention [19]. Another study aimed to identify the most consistently used definitions of “flexitarian” dietary patterns, or dietary patterns with a reduced amount of animal foods, which are related to low environmental impact [20]. This study suggested that following a flexitarian dietary pattern in terms of reducing or limiting red meat may be feasible and even implicitly recommended by the official dietary guidance of several countries. Moreover, it was found that most food-based dietary guidance (FBDGs) examined did not include recommendations to decrease dairy or fish intake [20]. A cross-sectional, nationwide study examined the eating habits of a representative sample of Italian university students to identify the determinants of adherence to the Mediterranean Diet (MD) and the most relevant actions to improve their well-being [21]. The Mediterranean Diet Quality Index for Children and Adolescents (KIDMED) and Sustainable Healthy Diet (SHED) index questionnaires were used to explore MD adherence as the primary outcome and dietary behavior sustainability, respectively. This study showed that having an active lifestyle, eating more plant products, and having more sustainable dietary behaviors in terms of the SHED index were the main determinants of a high KIDMED score [21].

It is currently well-recognized that the personalized nutrition programs enhanced with artificial intelligence (AI)-based tools can hold promising potential for the development of healthy and sustainable diets and for disease prevention. In this aspect, a study by Rouskas et al. aimed to explore the impact of an AI-based personalized nutrition program on the gut microbiome of healthy individuals [22]. This study provided evidence for a beneficial effect of an AI-based personalized nutrition program aimed at increasing MD adherence on the gut microbiome composition of healthy individuals [22]. Another cross-sectional, nationwide study investigated the association between ultra-processed foods (UPFs) consumption and plant-based diet quality in Australian adults [23]. This study supported substantial evidence that higher consumption of UPFs was associated with a lower plant-based diet quality. Notably, these findings may have implications for the design of dietary interventions that encourage the consumption of minimally processed plant-based foods, which are related to lower environmental impacts [23]. Moreover, a review article explored current knowledge on the environmental, oxidative, and genomic effects of sucralose (E955), an artificial sweetener widely used in food products, including those for children, and known to cross both the placental barrier and into breast milk [24]. Structurally similar to chlorinated compounds such as perfluoralkyl substances (PFAS), sucralose is highly persistent in the environment, which complicates its degradation and removal, especially from aquatic systems. Thus, from an environmental perspective, the persistence of sucralose in aquatic and terrestrial ecosystems presents an ongoing ecological challenge [24]. Finally, balancing consumer demand for low-calorie sweeteners with health and environmental safety requires a comprehensive understanding of compounds like sucralose, combined with proactive strategies for monitoring, mitigation, and substitution. This integrated approach will contribute to more sustainable food systems and healthier ecosystems in the long term [24].

Conclusively, ongoing research supports significant evidence that sustainable diets may provide multiple beneficial effects in both human and planet health [25,26]. There are preliminary data that flexitarian diets and territorial diversified diets have a greater impact on the environment than vegan, vegetarian, and pescatarian diets; however, the negative effects are considerably reduced compared with Western diets, especially if diets include locally sourced seasonal foods [25,26]. Alarming enough, the currently existing literature remains limited, highlighting the need for performing both cross-sectional and longitudinal clinical studies. The development and standardization of suitable questionnaires to assess sustainable diet adherence is recommended. Plant-based diets with an absence or minimal consumption of animal food products and UPFs should be performed in humans by the use of a reliable, “gold standard” sustainable diet index [4,12,27,28,29]. Moreover, future studies should be focused on defining more precisely optimal sustainable healthy diets for different populations, ensuring that such diets are sufficiently affordable and accessible to people in all countries.
